# Soil *pqqC*-harboring bacterial community response to increasing aridity in semi-arid grassland ecosystems: Diversity, co-occurrence network, and assembly process

**DOI:** 10.3389/fmicb.2022.1019023

**Published:** 2022-10-21

**Authors:** Mei Zhang, Ruixi Zhang, Riquan Song, Xilong An, Guixin Chu, Hongtao Jia

**Affiliations:** ^1^College of Grassland Science, Xinjiang Agricultural University, Urumqi, China; ^2^School of Life Science, Shaoxing University, Shaoxing, China; ^3^Inner Mongolia Autonomous Region Water Conservancy and Hydropower Survey and Design Institute Co., Ltd., Hohhot, China; ^4^Inner Mongolia Institute of Water Conservancy Science Research, Hohhot, China; ^5^Xilin Gol League Bureau of Agriculture and Animal Husbandry, Xilinhot, China; ^6^Xinjiang Key Laboratory of Soil and Plant Ecological Processes, Urumqi, China

**Keywords:** aridity, *pqqC* community, soil microorganisms, stochastic processes, network analysis, grassland ecosystems

## Abstract

Aridity is increasing in several regions because of global climate change, which strongly affects the soil microbial community. The soil *pqqC*-harboring bacterial community plays a vital role in soil P cycling and P availability. However, the effect of shifts in aridity on the *pqqC* community is largely unknown. Here, based on high-throughput sequencing technology, we investigated the response patterns of the diversity, co-occurrence networks, and assembly mechanisms of the soil *pqqC* communities along a natural aridity gradient in adjacent pairs of natural and disturbed grasslands in Inner Mongolia, China. The results showed that the α-diversity of the *pqqC* community first increased and then decreased with increasing aridity in the natural grassland, while it linearly increased as aridity increased in the disturbed grassland. The *pqqC* community dissimilarity significantly increased with increased aridity, exhibiting a steeper change rate in the disturbed grassland than in the natural grassland. Increased aridity altered the *pqqC* community composition, leading to increases in the relative abundance of *Actinobacteria* but decreases in *Proteobacteria*. The composition and structure of the *pqqC* community showed significant differences between natural and disturbed grasslands. In addition, the network analysis revealed that aridity improved the interactions among *pqqC* taxa and promoted the interspecific competition of *pqqC* microorganisms. The *pqqC* community assembly was primarily governed by stochastic processes, and the relative contribution of stochastic processes increased with increasing aridity. Furthermore, disturbances could affect *pqqC*-harboring bacterial interactions and assembly processes. Overall, our findings fill an important knowledge gap in our understanding of the influence of aridity on the diversity and assembly mechanism of the soil *pqqC* community in grassland ecosystems, and this work is thus conducive to predicting the *pqqC* community and its ecological services in response to future climate change.

## Introduction

The frequency and severity of extreme drought events will continue to increase due to global climate change (IPCC, 2013; Asadi Zarch et al., 2017), and these changes will threaten the functioning of terrestrial ecosystems (Bradford et al., 2020). Numerous studies have reported that increased aridity significantly affects belowground communities, such as by reducing plant biomass, suppressing plant growth, and altering plant traits and community diversity (Luo et al., 2020; Ma et al., 2022). In contrast, our understanding of aridity-induced changes in belowground soil microbial communities is limited. Soil microorganisms are an indispensable component of ecosystems and play a crucial role in maintaining the functions and services of terrestrial ecosystems, such as soil carbon sequestration, nutrient cycling, soil fertility promotion, and plant productivity (Bardgett and van der Putten, 2014; Kou et al., 2021). Therefore, it is imperative to explore the responses of soil microbes to increased aridity in order to predict changes in ecosystem functions and services under future climate change scenarios.

Recently, a meta-analysis showed that aridity decreased the diversity of soil bacterial and fungal communities at the global scale (Yang et al., 2021). Meanwhile, some studies have pointed out that the diversity, structure, and community of soil microorganisms could be directly or indirectly affected by aridity (Wang et al., 2015; Chen et al., 2022). For example, Maestre et al. (2015) found that aridity indirectly reduced soil bacterial and fungal community diversity by decreasing the soil organic carbon content in global drylands. Similarly, Huang et al. (2019) noted that the richness and composition of soil archaeal community were indirectly affected by aridity as aridity enhanced the soil electrical conductivity and reduced the soil total nitrogen content at a regional scale. Although some studies have been carried out to investigate the influences of aridity on soil microorganisms (bacteria, fungi, and archaea), the changes that occur in soil functional microbes along an aridity gradient are poorly understood. Nowadays, only Zhou et al. (2021) reported the patterns of N functional genes abundances along the natural precipitation in Inner Mongolia.

Phosphorus (P) is an essential nutrient element for plant growth (Khan et al., 2010), and its availability is vital for the productivity of croplands and natural ecosystems. Moreover, microbes play crucial roles in soil P cycling and the regulation of soil P availability. The main mechanisms for the increases in soil P availability by soil microorganisms include: (i) mineralizes soil organic P into plant-available inorganic P by secreting phosphatases (George et al., 2018) and (ii) solubilizes insoluble inorganic P by extruding protons and producing organic acids such as gluconic acid, citric acid, and oxalic acid (Kour et al., 2021). Recently, the soil *pqqC* (pyrroloquinoline-quinone synthase C) gene is typically used as an effective marker gene for inorganic P-solubilizing bacteria, and it contributes to the production of gluconic acid (Meyer et al., 2011). However, significant knowledge gaps remain regarding how aridity affects the diversity and composition of the soil *pqqC* community.

Nowadays, unraveling the mechanisms of the microbial community assembly is a central issue in microbial ecology (Nemergut et al., 2013; Chu et al., 2020). Ecologists have proposed that both deterministic processes (e.g., environmental filtering and biotic interactions) and stochastic processes (e.g., ecological drift, dispersal, and extinction/speciation) jointly govern the microbial community assembly (Hubbell, 2001; Fargione et al., 2003; Chave, 2004; Stegen et al., 2012). Recent studies have reported that the assembly mechanisms of soil diazotrophs, nirK-type and nirS-type denitrifiers, and bacteria were predominantly driven by deterministic processes along the elevational gradient (Li et al., 2018; Wang et al., 2019; Kou et al., 2021). Deterministic processes were found to be the primary drivers of the soil fungal community assembly in the 0-10-cm soil layer in paddy fields, while stochastic processes predominated in shaping the fungal community in the 20-40-cm soil layer (Li et al., 2020). A recent study also revealed that aridity was the primary factor influencing the soil bacterial community assembly (Pan et al., 2022). In addition, network analyses based on significant correlations have been extensively employed to infer the interactions among microbes in various environments (Ma et al., 2016; Pan et al., 2022), thus contributing to our understanding of the structure and assembly of soil microbial communities (Barberán et al., 2012; Hunt and Ward, 2015). Nevertheless, how aridity affects the co-occurrence networks and assembly mechanisms of the soil *pqqC* community along a natural aridity gradient remains unclear.

Grasslands cover approximately 40% of the Earth’s surface and provide a series of ecosystem services such as food production, carbon storage, nutrient cycling, and climate mitigation (Bardgett et al., 2021). The semi-arid grasslands on the Inner Mongolian Plateau as a typical representative ecosystem of the Eurasian steppe are extremely vulnerable to climate change (Bai et al., 2012). This region provides an ideal experimental platform for elucidating the influences of aridity on soil microbes due to the existence of an east–west aridity gradient (Bai et al., 2008). In addition, Inner Mongolian grasslands often encounter various anthropogenic disturbances such as grazing, mowing, and reclamation, and these influences may alter the soil bulk density, moisture content, and nutrient contents. Given the differences in soil properties between natural and disturbed grasslands, these disturbances may alter the pattern of the soil *pqqC* community in response to increasing aridity. Therefore, in this work, we conducted a small-scale investigation of the *pqqC* community in adjacent pairs of natural and disturbed grasslands across an east–west natural aridity gradient in Inner Mongolia, China. The specific objectives were to (i) explore the response pattern of the diversity and composition of soil *pqqC* community to increasing aridity in natural and disturbed grasslands, (ii) clarify how aridity affects *pqqC* community assembly processes, and (iii) assess the co-occurrence patterns of the *pqqC* community in response to increasing aridity.

## Materials and methods

### Site description and field sampling

**Figure 1:**
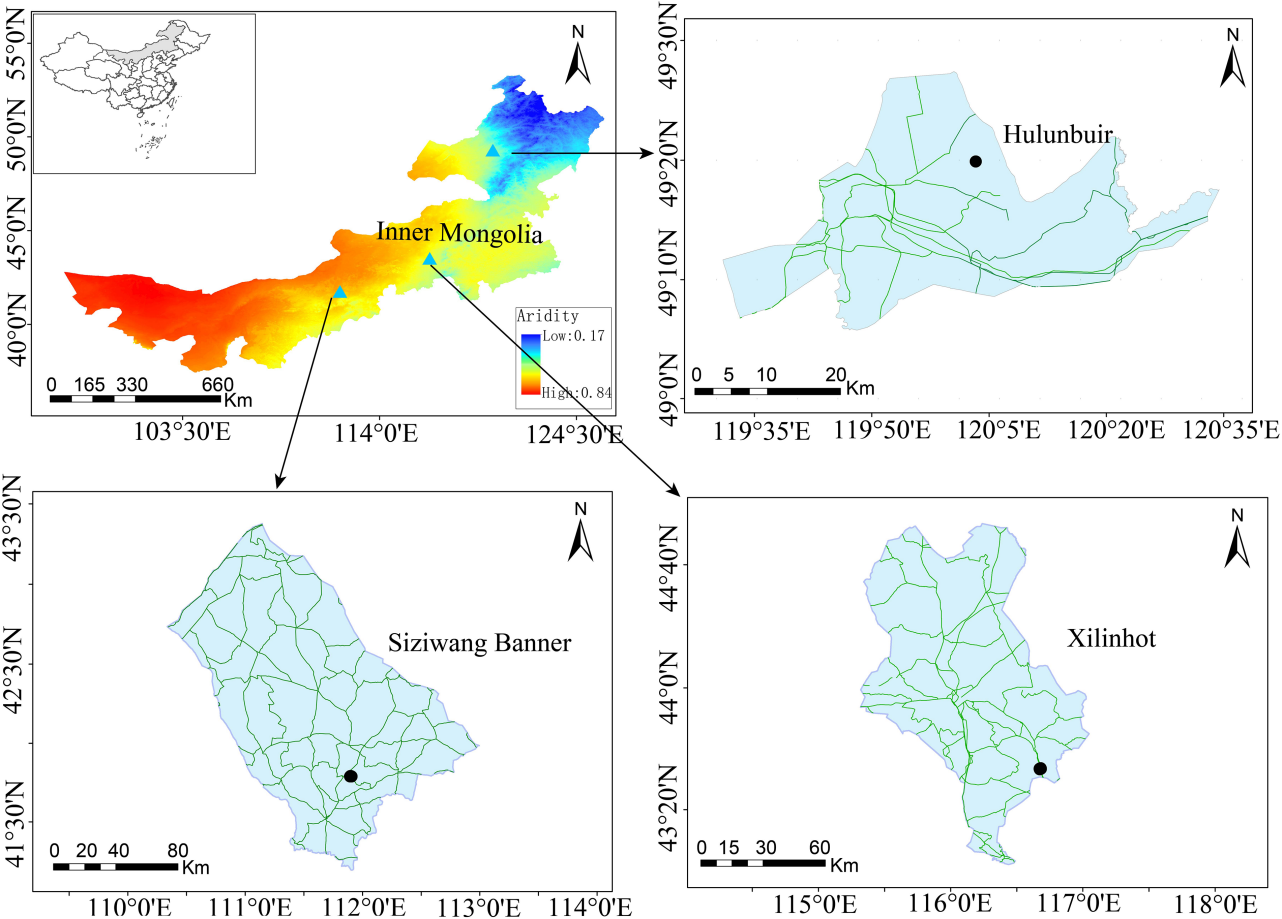
Study areas and sampling sites (Aridity is presented as one minus the aridity index).

This study was performed across an east-to-west precipitation transect in the Inner Mongolia Autonomous Region, China (97°12′ to 126°04′E, 37°24′ to 53 °23′N). This area belongs to a semi-arid continental climate. Soil samples were collected in mid-July 2019 from three sites (i.e., Hulunbuir, Xilinhot, and Siziwang Banner) along this transect (Figure 1). Soil samples from these three sites can effectively represent the change in the aridity gradient in Inner Mongolia grasslands, and the sites were artificially divided into low-aridity level, medium-aridity level, and high-aridity level, respectively. The soil types, vegetation types, and dominant plants at each site are listed in Table 1.

**Table 1:** Descriptions of soil classifications, vegetation types, and dominant plant species for each site in this study.

Sites	Soil types	Vegetation types	Dominant plant species
Hulunbuir	*Haplic Chernozems*	Meadow steppe	*Stipa baicalensis, Leymus chinensis*, *Carex duriuscula*, *Artemisia tanacetifolia*, *Cleistogenes squarrosa*, *Pulsatilla turczaninovii*
Xilinhot	*Haplic Kastanozems*	Typical steppe	*Stipa grandis*, *Leymus chinensis*, *Agropyron michnoi*, *Cleistogenes squarrosa*, *Carex korshinskyi*, *Potentilla acaulis*
Siziwang Banner	*Calcic Kastanozems*	Desert steppe	*Stipa breviflora*, *Artemisia frigida*, *Cleistogenes songorica*, *Kochia prostrata*, *Salsola collina*

The soil classification according to the FAO soil taxonomy.

For each site, two similar treatments (i.e., natural grassland and disturbed grassland) were established, and triplicate sample replicates were performed for each treatment. The plots of the two treatments were adjacent at each site. Full details of the experimental treatment for each site are listed in Supplementary Table S1. Overall, 18 samples were collected with triplicates for each treatment from each site (3 sites × 2 treatments × 3 replicates). Specifically, a 100-m belt transect was randomly established at each plot (each treatment replicate). Afterward, 10 soil core samples were taken from the topsoil (0–15 cm) along the belt transect and pooled as a single sample for subsequent analyses. All collected soil samples were sieved through 2-mm mesh and separated into two subsamples. The subsample intended for the analyses of soil physiochemical properties was air-dried, and the other intended for DNA extraction was stored at −80°C. The edaphic properties, including pH, NH_4_^+^, NO_3_^−^, soil organic carbon, total phosphorus, total nitrogen, and available phosphorus, were determined according to the standard testing methods described by Bao (2000). Soil moisture was determined gravimetrically after drying at 105°C for 24 h. In addition, a portable global positioning system (GPS) device was used to record the spatial geographic coordinates of each sample. The mean annual temperature (MAT) and mean annual precipitation (MAP) data were obtained from the global WorldClim database (Hijmans et al., 2005). The aridity index based on the ratio of precipitation and potential evapotranspiration was extracted from the Climate Database v2.^1^ Aridity is presented as one minus the aridity index in this study.

For the collection of plant samples, three 1-m × 1-m quadrats were randomly arranged in each plot. In each quadrat, the plant richness was determined by recording the total number of plant species. Afterward, the aboveground biomass was clipped from the ground surface in each quadrat, and weighed to determine the plant biomass after drying at 65°C for 48 h. The climate data, geographical variables, soil properties, and plant attributes mentioned above for each sample are given in Supplementary Table S2.

### Soil DNA extraction, PCR amplification, and Illumina sequencing

Total soil DNA was extracted from 0.25 g of soil using a PowerSoil® DNA Isolation Kit for soil (MoBio, Carlsbad, CA, United States) following the manufacturer’s instructions. The quality and quantity of the extracted DNA were tested using a NanoDrop ND-1000 spectrophotometer (Thermo, USA), and the extracted soil DNA was stored at −20°C for subsequent analyses.

The amplification of the *pqqC* gene was carried out using the paired primers of pqqC-Fw AACCGCTTCTACTACCAG and pqqC-Rv GCGAACAGCTCGGTCAG (Bi et al., 2020). The reaction system contained 0.4 μl forward primer (10 μM), 0.4 μl reverse primer (10 μM), 1 μl DNA template, 10 μl of 2 × EasyTaq polymerase chain reaction (PCR) SuperMix, and 10 μl double distilled water. The amplification protocol involved an initial denaturation for 5 min at 95°C, followed by 40 amplification cycles (95°C for 15 s, 60°C for 30 s). The amplification efficiency was 81.00%, and the R^2^ value was >0.998.

The PCR products were sequenced on the Illumina MiSeq platform according to the standard protocols at Shanghai Personal Biotechnology Co., Ltd., Shanghai, China. The raw sequences were analyzed by QIIME 2 software (Quantitative Insights into Microbial Ecology 2) and the Vsearch pipeline (v2.13.4; Rognes et al., 2016; Bolyen et al., 2019). After quality filtering and chimera detection, the remaining high-quality sequences were clustered with a 97% similarity threshold, and assigned to operational taxonomic units (OTUs). Subsequently, the taxonomic identification of each OTU was performed using BLAST by the local nt database.

### Statistical analysis

The *t*-test was performed to compare the α-diversity difference between the *pqqC* communities in the natural and disturbed grassland by SPSS 21.0 Statistics (ISPSS Inc., Chicago, IL, United States). The normality of all data was checked using the Shapiro–Wilk test. The vegan package was employed to calculate the α-diversity, including Chao1 index and OTU richness, in R software (version 4.0.3). An ordinary least-squares regression model was utilized to evaluate the correlations between aridity and *pqqC* community α-diversity, *pqqC* community β-diversity (Bray–Curtis dissimilarity), and the dominant phyla and genera of the *pqqC* community. The β-diversity of the *pqqC* community was determined by using the first axes identified from the principal co-ordinates analysis (PCoA) results. Redundancy analysis (RDA) was used to identify the main environment variables affecting the variations of soil *pqqC* community structure through CANOCO 5.0 (Microcomputer Power, Ithaca NY, USA). The normalized stochasticity ratio (NST) was calculated to explore the assembly processes of the soil *pqqC* community as aridity increased by using the “NST” package. NST values below or above the 50% boundary point represent the deterministic or stochastic processes governing *pqqC* community, respectively. Moreover, a neutral community model was applied to further confirm the potential contribution of stochastic processes to the *pqqC* community assembly with increasing aridity (Sloan et al., 2006; Chen et al., 2019). A co-occurrence networks analysis was performed to assess the complexity of the *pqqC* community by Gephi software 0.9.2. To reduce the dataset complexity, only OTUs with average relative abundances >0.01% were selected for the subsequent analysis. A Pearson’s correlation matrix between *pqqC* OTUs was calculated using the “psych” and “WGCNA” packages, and only Pearson’s correlation coefficients >0.6 and *p* values <0.01 were selected for the construction of networks. Finally, the topological parameters of the networks, including the nodes, edges, clustering coefficient, modularity, and average degree, were calculated using Gephi software 0.9.2. A random forest analysis was carried out to assess the important predictors affecting the α-diversity of the *pqqC* community by using the “randomForest,” “rfPermute,” and “A3” packages. To further explore the direct and indirect effects of aridity, plant variables, and soil properties on the variations of α-diversity and composition of the soil *pqqC* community, partial least squares path modeling (PLS-PM) was performed using the “plspm” package in R.

## Results

### The diversity of the *pqqC* community

**Figure 2:**
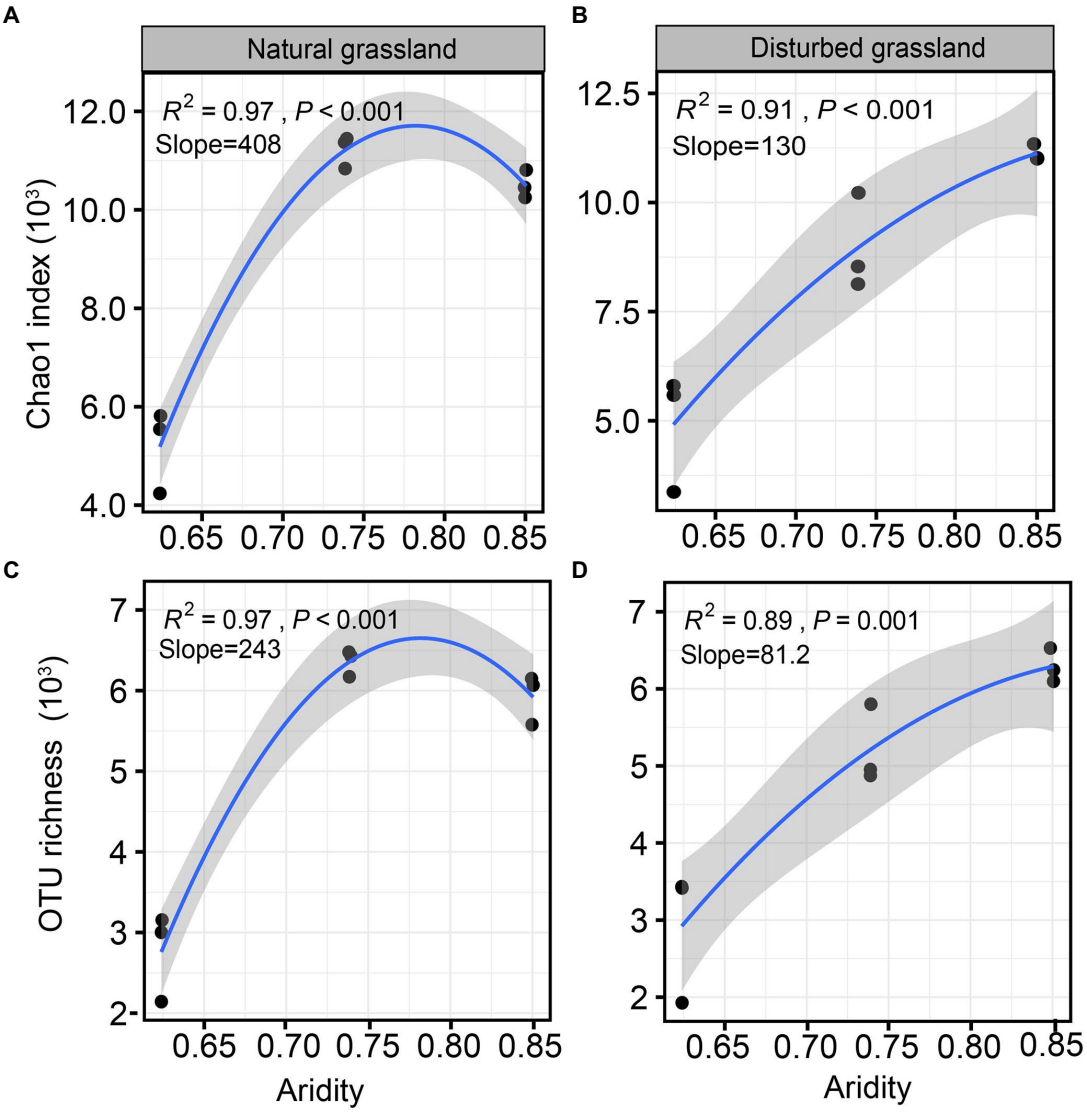
Relationships between aridity and α-diversity indexes (Chao1 index and OTU richness) of soil *pqqC* community in natural **(A,C)** and disturbed **(B,D)** grasslands.

**Figure 3:**
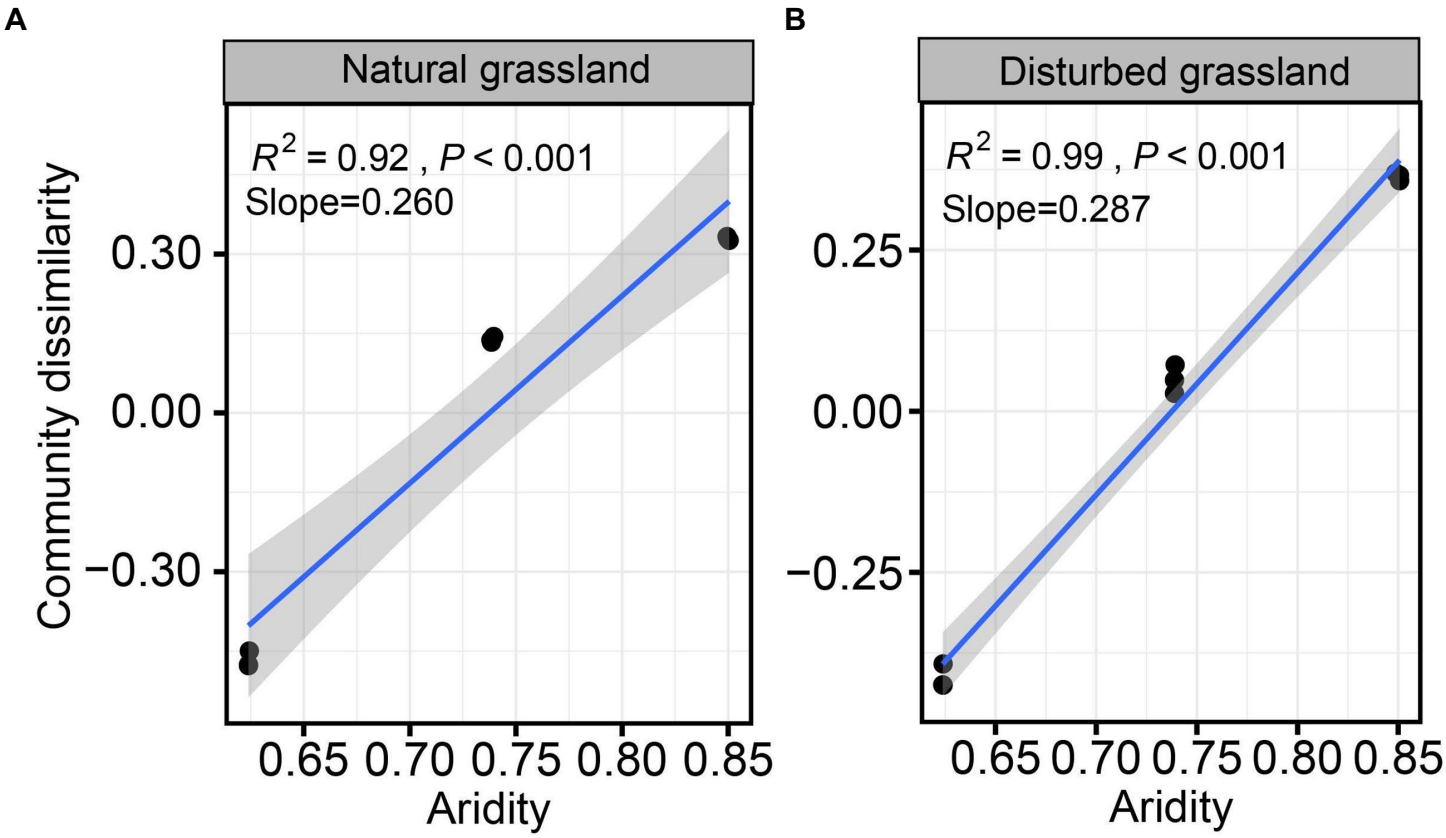
Relationships between aridity and soil *pqqC* community dissimilarity (β-diversity) in natural **(A)** and disturbed **(B)** grasslands.

The diversity of the *pqqC* community was significantly affected by increasing aridity, and disturbance altered the diversity pattern of the *pqqC* community along the aridity gradients (Figure 2). For instance, the Chao1 index of the *pqqC* community showed a significant nonlinear relationship with increasing aridity in both natural and disturbed grasslands, and exhibited the greater R^2^ and slope values in the natural grassland (R^2^ = 0.97, Slope = 408) than that in the disturbed grassland (R^2^ = 0.91, Slope = 130; Figures 2A,B). A similar tendency was observed for the OTU richness of the *pqqC* community (Figures 2C,D). Besides, the soil *pqqC* community dissimilarity drastically increased as increasing aridity, and a greater change rate was obtained in the disturbed grassland (slope = 0.260) than in the natural grassland (slope = 0.287; Figure 3).

### The structure and composition of the *pqqC* community

**Figure 4:**
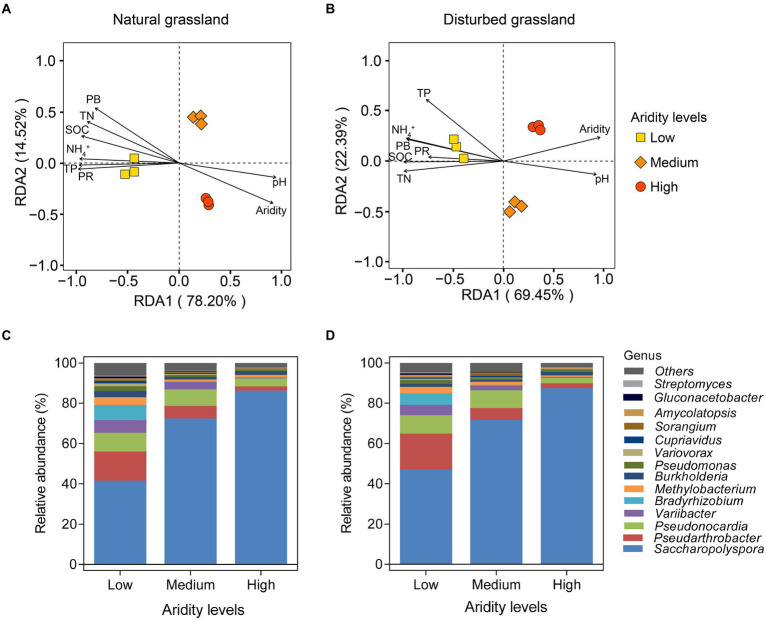
Redundancy analysis (RDA) of soil *pqqC* community and environment variables under different aridity levels in natural **(A)** and disturbed **(B)** grasslands. SOC, soil organic carbon; TN, total nitrogen; TP, total phosphorus; PB, plant biomass; PR, plant richness. Relative abundances of the *pqqC* community at genus level (the top 15 genera) under different aridity levels in natural **(C)** and disturbed **(D)** grasslands.

The RDA analysis showed a clear separation of the *pqqC* community among different aridity gradients in the natural grassland (Figure 4A). All of the samples were separated into three groups based on aridity, i.e., low-, medium-, and high-aridity levels. Similar effects were observed in the disturbed grassland (Figure 4B). Furthermore, in typical steppe, the significant environment variables affecting the soil *pqqC* community structure followed the sequence: aridity (*r*^2^ = 0.993, *p* = 0.001) > SOC (*r*^2^ = 0.975, *p* = 0.001) > PR (*r*^2^ = 0.973, *p* = 0.01) > TN (*r*^2^ = 0.969, *p* = 0.002) > PB (*r*^2^ = 0.962, *p* = 0.001) > TP (*r*^2^ = 0.955, *p* = 0.012) > NH_4_^+^ (*r*^2^ = 0.945, *p* = 0.016) > pH (*r*^2^ = 0.912, *p* = 0.007; Figure 4A); similar, environment variables significantly affected the soil *pqqC* community structure in desert steppe, which included aridity > TN > NH_4_^+^ > SOC > TP > PB > pH > PR (Figure 4B). Besides, all *pqqC* gene sequences in our soil samples were affiliated with 4 phyla and 73 genera. The dominant phyla included *Actinobacteria* (average relative abundance of 83.8%), *Proteobacteria* (average relative abundance of 14.7%), and *Nitrospirae* (average relative abundance of 0.02%; Supplementary Figure S1). *Saccharopolyspora*, *Pseudarthrobacter*, *Pseudonocardia*, *Variibacter*, and *Bradyrhizobium* were the dominant genera and together accounted for an average of 88.30% of all sequences (Figures 4C,D). Aridity significantly affected the major phyla and genera, and similar distribution patterns were found between the natural and disturbed grasslands along the aridity gradients. Specifically, the relative abundance of the phylum *Proteobacteria* declined linearly with increasing aridity, while *Acidobacteria* showed the opposite pattern (Supplementary Figures S2A–C). The relative abundance of *Nitrospirae* was nonlinearly related to aridity. Furthermore, seven genera, including *Pseudarthrobacter*, *Pseudonocardia*, *Variibacter*, *Bradyrhizobium*, *Methylobacterium*, *Pseudomonas*, and *Variovorax*, significantly decreased as aridity increased, while the relative abundance of the genus *Saccharopolyspora* exhibited the opposite tendency (Supplementary Figures S2D–L). Aridity had no significant impact on the genus *Burkholderi*a (*p* > 0.05).

### Co-occurrence network analysis

**Figure 5:**
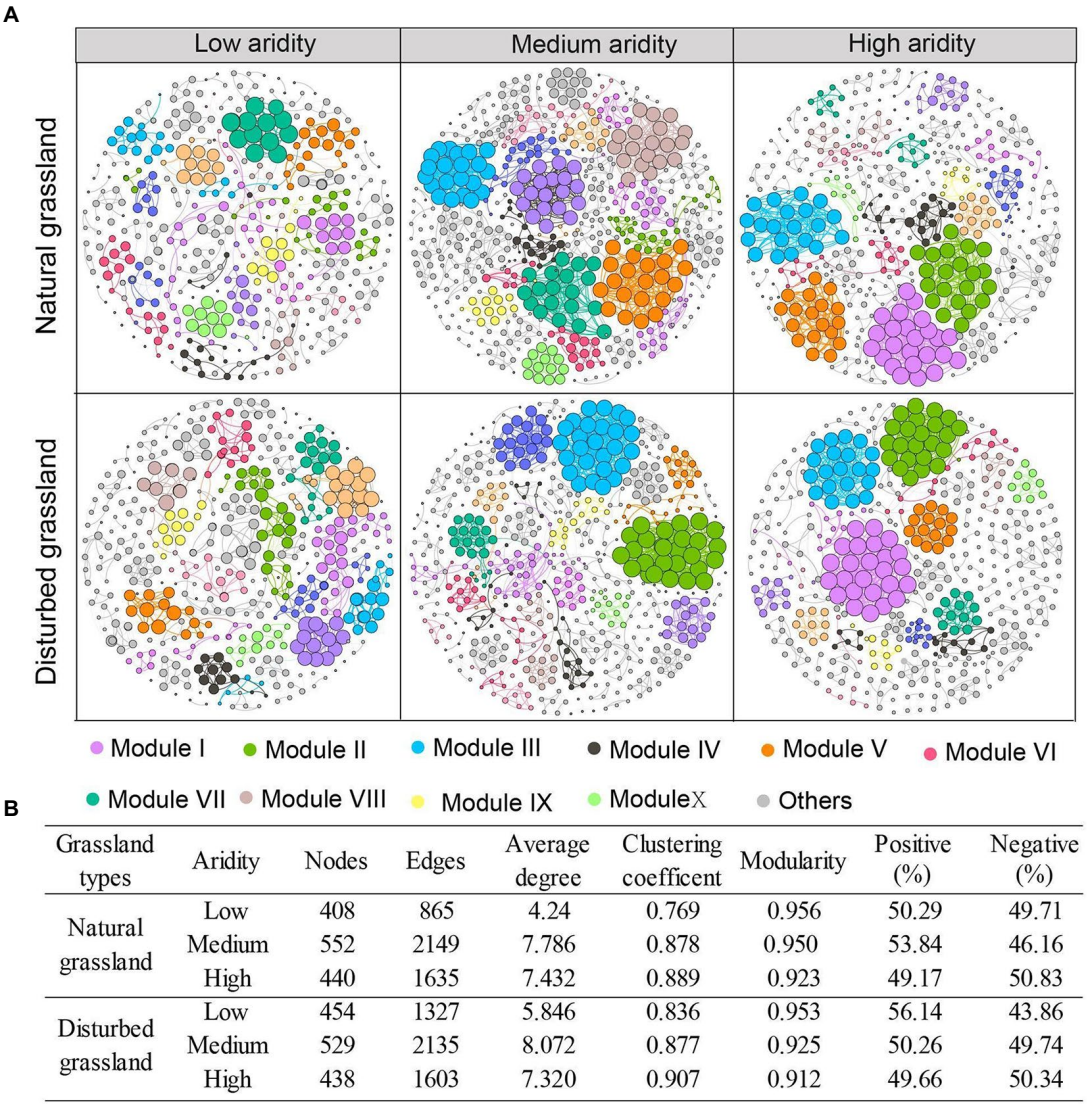
Co-occurrence patterns of *pqqC* community under different aridity levels in natural and disturbed grasslands **(A)**. Nodes represent individual OTUs; edges represent significant strong and significant Spearman correlations (r > 0.6, *p* < 0.01). Major modules are shown in different colors, and smaller modules are shown in grey. The size of each node is proportional to the degree. The variations of topological features of networks under different aridity levels in natural and disturbed grasslands **(B)**.

The network complexity of the *pqqC*-encoding community was significantly impacted by aridity (Figure 5). In both the natural and disturbed grasslands, aridity increased the topological parameters, including the node number, edge numbers, and average degree, and the highest values of these terms were found at the medium-aridity level (Figure 5B). The clustering coefficient increased with increasing aridity, while modularity exhibited an opposite tendency. Moreover, the percentage of positive associations decreased from 50.29 to 49.17% as the aridity increased in the natural grassland, and decreased from 56.14 to 49.66% in the disturbed grassland. These results indicated that a relatively higher aridity stress improved the network complexity and competition relationships among *pqqC*-harboring bacteria. Besides, the node number, edge numbers, average degree, clustering coefficient, and proportion of positive connections were higher in the disturbed grassland than in the natural grassland (Supplementary Figure S3; Supplementary Table S3).

### Assemblage processes of the *pqqC* community

**Figure 6:**
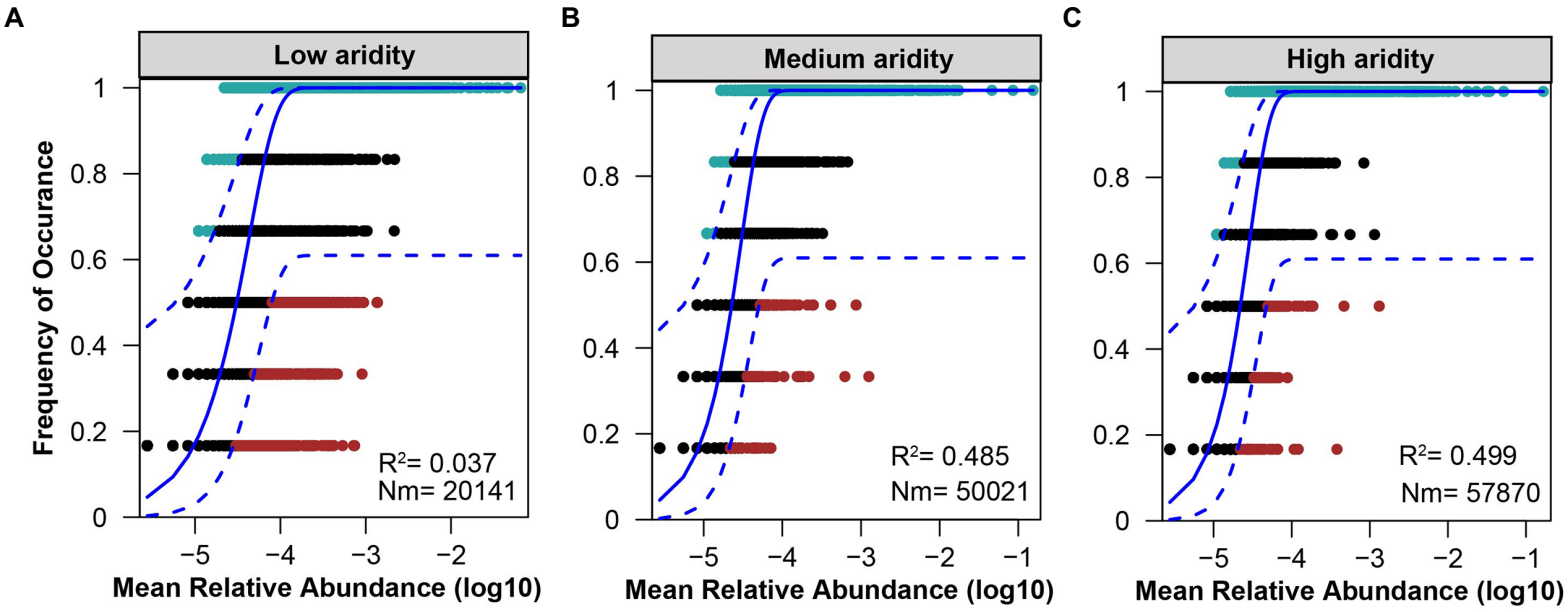
Fit of the neutral community model (NCM) of *pqqC* community in the low **(A)**, medium **(B)**, and high **(C)** aridity levels. The solid blue lines indicate the best ft to the neutral model and the dashed blue lines represent 95% confdence intervals around the model prediction. OTUs that occur more frequently than predicted by the model are shown in aquamarine, while those that occur less frequently than predicted are shown in red. OTUs that occur within prediction are shown in black. Rsqr indicates the goodness of ft to the neutral model. Nm indicates the metacommunity size times immigration.

**Figure 7:**
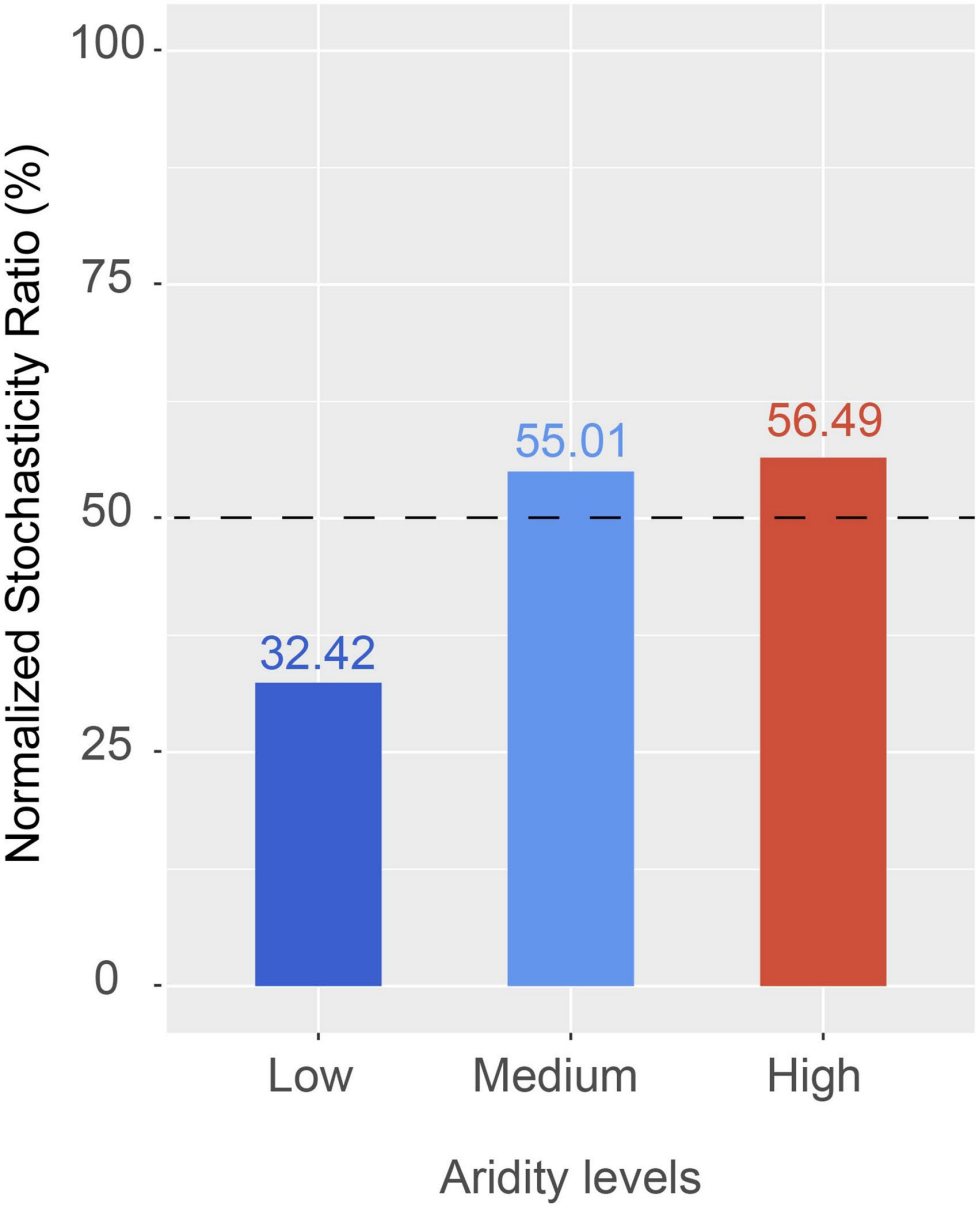
The normalized stochasticity ratio (NST) of soil *pqqC* community in different aridity levels.

The ecological processes of *pqqC* community assembly were determined by using the neutral community model and normalized stochasticity ratio (NST). As shown in Supplementary Figure S4, the neutral community model showed that the R^2^ value based on all of the samples was 0.244 (above zero), implying that the *pqqC* community assembly fit the neutral model. Moreover, the relative importance of stochastic processes increased gradually with increasing aridity, explaining 3.7, 48.5, and 49.9% of the *pqqC* community variance at the low-, medium-, and high-aridity levels, respectively (Figure 6). In addition, the NST value based on all of the samples was 65.73% (above the 50% boundary point), suggesting that the *pqqC* community was predominantly controlled by stochastic processes (Supplementary Figure S5). Furthermore, the NST value increased from 32.42 to 56.49% with increasing aridity, suggesting that the relative influence of stochastic process associated with the *pqqC* community assembly was strengthened as aridity increased (Figure 7). Overall, these results suggested that stochastic processes, rather than deterministic processes, dominated the *pqqC* community assembly and that the relative importance of stochastic processes increased with increasing aridity.

## Discussion

### Effects of increasing aridity on the diversity and composition of *pqqC* community in natural and disturbed grasslands

**Figure 8:**
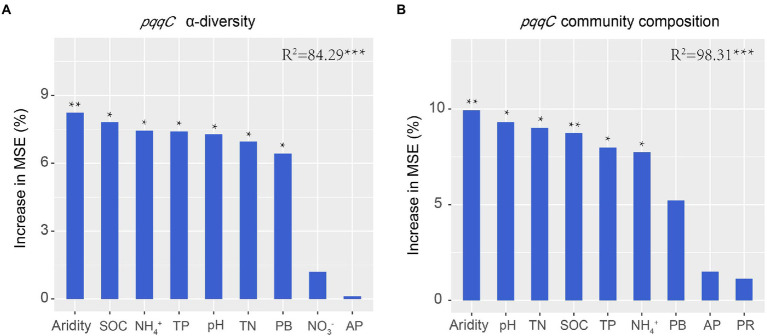
Random forest analysis shows the main predictors for the α-diversity **(A)** and composition **(B)** of *pqqC* community. TN, total nitrogen; SOC, soil organic carbon; TP, total phosphorus; PB, plant biomass; AP, available phosphorus; PR, plant richness.

Previous studies have demonstrated that soil biotic community is vulnerable to drought in dryland ecosystems (Wang et al., 2015; de Vries et al., 2018; Berdugo et al., 2020). In the present study, the results of the random forest analysis and RDA demonstrated that aridity was the most important factor affecting *pqqC* community α-diversity (Figures 4, 8). Our data showed that the *pqqC* community α-diversity first increased and then decreased with increasing aridity in the natural grassland (Figure 2); this finding was consistent with reports of nematode communities (fungal feeders and omnivores/predators) in semi-arid grassland ecosystems of northern China when aridity was between 0.6 and 0.9 (Xiong et al., 2019). In contrast, the α-diversity of the *pqqC* community in the disturbed grassland increased with increasing aridity. In line with this, Pan et al. (2022) reported that the α-diversity of soil bacterial community in agricultural ecosystems increased as aridity increased when aridity was below 0.92. Other studies also reported that the Shannon index of soil bacterial community was positively associated with aridity in a tallgrass prairie ecosystem in the United States (Fierer et al., 2013). Nevertheless, numerous studies have shown that increasing aridity reduces the α-diversity of soil archaea, fungi, and bacteria at large scales (Huang et al., 2019; Wang et al., 2021). These contrary observations may be attributable to (i) the differences in microbial community composition among different habitats (Liu N. et al., 2022); (ii) low-aridity levels between 0.6 and 0.9 in our study, potentially being an intermediate disturbance to soil microorganisms (Molino and Sabatier, 2001); thus, increased aridity positively impacted the a-diversity of the *pqqC* community; or (iii) temperature being gradually improved as aridity increased in our study area, resulting in an increased soil microbial richness (Wang et al., 2016).

In addition, our results showed that the *pqqC* community dissimilarity was significantly positively correlated with increasing aridity, indicating that the greater difference in aridity between two sites is, the greater dissimilarity of the *pqqC* community between the two sites is. This pattern is similar to that found in soil bacterial communities in different types of ecosystems, such as grassland, forest, farmland, desert, and wetland, at large scales (Pan et al., 2022). Interestingly, we found that the change rate of the *pqqC* community dissimilarity along the aridity gradient was higher in the disturbed grassland than that in the natural grassland, implying disturbance weakened the dispersal rate of *pqqC*-harboring bacteria. This finding may strongly relate to environmental variabilities and spatial heterogeneity (Nekola and White, 1999; Pan et al., 2022). Long-term disturbances could alter soil properties and plant attributes such as soil moisture, nutrient contents, and plant biomass (Supplementary Table S2). In this case, as environmental differences increase, compositional spatial variation became more pronounced, thus strengthening such a relationship (Wang et al., 2017). On the other hand, differences in spatial configurations or contexts, such as the sizes or isolation of habitats in different ecosystems, could further affect the movement and dispersal abilities of soil microorganisms, and ultimately result in the different change rates of *pqqC* community dissimilarity between the natural and disturbed grasslands (Tuomisto et al., 2003; Wang et al., 2017).

**Figure 9:**
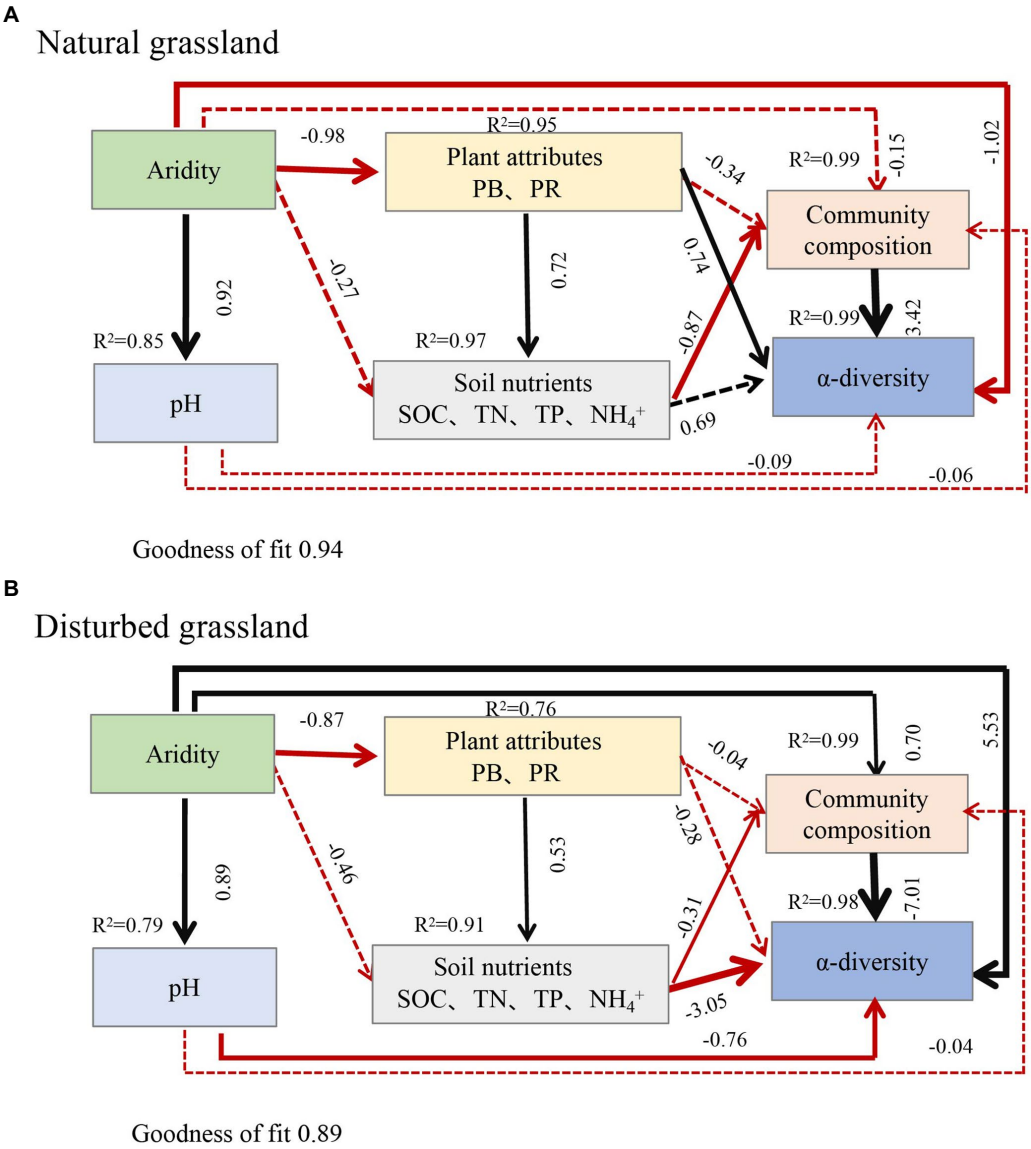
Partial least squares path modelling (PLS-PM) disentangling the direct and indirect influence of aridity on the composition and α-diversity of soil *pqqC* community in natural grassland **(A)** and disturbed grassland **(B)**. Black arrows and red arrows denote significantly positive and negative relationships, respectively. Solid and dashed arrows represent significant and non-significant paths, respectively. Number above each arrow represents standardized path coefficients (the effect size of the relationship). The width of the arrows indicates the strength of the standardized path coefficient. R^2^ indicates the variance of dependent variable explained by the model. PB, plant biomass; PR, plant richness; SOC, soil organic carbon; TN, total nitrogen; TP, total phosphorus.

Aridity was also the key factor explaining the variations in soil *pqqC* community composition (Figure 8). In the present study, aridity significantly affected the dominance of major phyla and genera in both natural and disturbed grasslands. For example, the relative abundance of the phylum *Proteobacteria* declined linearly with increasing aridity, while *Actinobacteria* exhibited the opposite trend; these findings were consistent with previous observations (Wang et al., 2015; Yao et al., 2017). Moreover, the reduced *Proteobacteria* abundance was largely driven by decreases in some genera, including *Variibacter*, *Bradyrhizobium*, *Methylobacterium*, *Pseudomonas*, and *Variovorax,* while the changes in *Actinobacteria* were mainly attributed to increases in *Saccharopolyspora. Actinobacteria* are oligotrophic taxa with strong drought resistance due to their unique cell wall composition (Manzoni et al., 2012; Liu et al., 2019). Furthermore, the phylum *Actinobacteria* has a strong ability to hydrolyze complex compounds, including pectin, cellulose, xylan, and starch (Favet et al., 2013), and this ability could help these bacteria to cope with deficient nutrient conditions. Therefore, the decreased soil water content and nutrient availabilities induced by drought could suppress the growth of copiotrophic microbes such as *Proteobacteria* (Delgado-Baquerizo et al., 2013; Yao et al., 2017), and this was supported by the PLS-PM results indicating that soil *pqqC* community composition could be indirectly affected by aridity due to the negative impacts of aridity on soil properties (Figure 9). In addition, despite the soil *pqqC* community composition patterns along the aridity gradients being similar between the natural grassland and the disturbed grassland, we found that disturbance significantly altered the structure and composition of the *pqqC* community at different aridity levels (Supplementary Figures S6, S7). Therefore, disturbance should be considered when predicting the consequences of increasing drought on soil *pqqC* communities and their ecological functions.

### Effects of increasing aridity on the co-occurrence patterns of the *pqqC* community in natural and disturbed grasslands

Complex interactions among microbial communities can be partially revealed by co-occurrence networks (Faust and Raes, 2012). In this study, a relatively high-aridity stress level increased the network complexity and interactions of *pqqC*-harboring bacteria. Nevertheless, our results were inconsistent with those of previous studies showing that drought may reduce the complexity and stability of the soil protistan network (Chen et al., 2022) or have no obvious influence on the soil fungal network (de Vries et al., 2018). These discrepancies may be attributed to i) different organisms have different tolerances to drought stress (Wu et al., 2018) and ii) different types of ecosystems with contrasting soil available nutrient contents and soil structures as well as vegetation coverage potentially resulting in differences in the sensitivity of soil microorganisms to drought stress (Soleimani et al., 2019). Generally, a positive or negative correlation reflects a cooperative or/and competitive interaction mode among different microorganisms in a co-occurrence network (Faust and Raes, 2012). A higher positive interaction in the microbial network indicates a cooperative relationship or mutualisms among different microorganisms, whereas a higher negative interaction represents antagonism. In this study, in both the natural and disturbed grasslands, the proportion of positive network correlations was higher than the proportion of negative correlations in the low-aridity level, whereas the high-aridity level showed the opposite trend, indicating that aridity stress prompted the transformation of interactions among *pqqC* taxa from cooperative to antagonistic. This may be because the greater soil *pqqC*-harboring bacterial biomass and population at high-aridity levels require sufficient nutrients to sustain their growth; however, the soil nutrient deficiencies that occur at high-aridity levels led to the increased interspecific competition (Ling et al., 2016). In addition, disturbances increased the network complexity and cooperative relationships among *pqqC* taxa (Supplementary Table S3), and this finding was attributed to *pqqC*-harboring bacteria defending themselves against harsh soil environmental conditions or poor P conditions by increasing their mutual connections (Wu et al., 2021). Some studies have reported that nutrient deficiencies and other environmental stresses in soils could enhance the trophic interactions and cooperation among soil microbes (Holtkamp et al., 2011; Hoek et al., 2016).

### Assembly mechanism of the soil *pqqC* community

A better understanding of how increasing aridity affects the assembly processes of soil microbial community is essential for clarifying the stability of biodiversity and ecosystem functions in response to future climate change (Chu et al., 2020). In the current study, soil *pqqC* community assembly on all of the samples was dominated by stochastic processes. In line with this, previous studies have shown that stochastic processes predominated the soil bacterial community assembly in the Namib Desert or the microeukaryotic community in lake ecosystems (Stomeo et al., 2013; Liu C. et al., 2022). Moreover, our results showed that the relative importance of stochastic processes on the *pqqC* community increased as aridity increased. This may be explained by the stronger stochastic dispersal of *pqqC* taxa under relatively high drought stress (Barberán et al., 2014). Besides, soil microbial community with a higher diversity was mainly driven by stochastic processes, but deterministic assembly processes were dominant in the low-diversity microbial community (Xun et al., 2019); this pattern was supported by our results regarding drought improved the α-diversity of the *pqqC* community (Figure 2). Recently, Chen et al. (2022) also showed that aridity increased the contribution of stochastic processes in shaping the protistan community. It is worth noting that disturbances reduced the relative importance of stochastic processes in governing *pqqC* community assembly (Supplementary Figures S4, S5), which was likely due to disturbance weakened the dispersal abilities of *pqqC*-harboring bacteria. Likewise, the changes in environmental conditions caused by disturbances could promote deterministic community assembly (Zhang et al., 2011).

### The indirect effects of aridity on soil *pqqC* communities *via* plant and soil variables

Numerous studies have showed that both edaphic factors (soil organic carbon inorganic N, and pH) and aboveground plant profiles (vegetation coverage, plant richness, and plant biomass) were important factors altering soil bacterial communities (Chen et al., 2015; Yao et al., 2017; Xu et al., 2020). In consistent with this, we found that SOC, TN, TP, NH_4_^+^, plant biomass, and plant richness notably affected the structure, composition, and diversity of the soil *pqqC* community (Figures 4A,B, 8). Further, our data showed that aridity was negatively related to these aforementioned factors (Figure 4), indicating that the alteration of *pqqC* community was presumably associated with the changes in these factors induced by aridity. Previous research elucidated that the decreases in coverage, plant richness, and aboveground biomass caused by aridity reduced organic C inputs and soil nutrient content (Delgado-Baquerizo et al., 2013; Berdugo et al., 2020) and accelerated soil N loss through denitrification (Evans and Burke, 2013). Based on the aforementioned studies, we further conducted PLS-PM analysis to explain how aridity, soil properties, and plant attributes mediate the changes in the composition and α-diversity of *pqqC* community. The results of PLS-PM evidenced that aridity indirectly altered the α-diversity and composition of the soil *pqqC* community *via* reducing plant biomass, plant richness, and soil nutrients in both natural and disturbed grasslands (Figures 9A,B). In agreement with our findings, Maestre et al. (2015) noted that the diversity and abundance of soil bacteria and fungi communities were associated with the negative effects of aridity on SOC and vegetation coverage. Moreover, previous findings showed that soil pH was the most important factor affecting the diversity and composition of the bacterial communities at a large scale (Rousk et al., 2010). However, we found that the influences of pH on the α-diversity and composition of *pqqC* community were not as significant as expected (Figures 9A,B). This was likely due to the relative narrow range of pH values in our study area (Maestre et al., 2015).

In addition, we must acknowledge that there are some other factors influencing *pqqC* community, with the exception of aridity. In other words, the effects of increasing aridity on *pqqC* community may likely be induced by the confounding effects including plant attributes, soil properties, and aridity itself. However, these effects currently are hardly separated individually. Therefore, an incubation experiment should be conducted to further investigate the patterns of the soil *pqqC* community in response to increasing aridity under different soil types in the future.

## Conclusion

In summary, aridity increased the α-diversity of the *pqqC* community and induced shifts in the composition of soil *pqqC* community. Aridity stress increased the network complexity and drove the transformation of interactions among the *pqqC* community from cooperation to antagonism. Aridity increased the importance of stochastic processes in governing the soil *pqqC* community assembly but decreased the importance of deterministic processes. Besides, disturbances changed the *pqqC* community diversity pattern as increasing aridity, *pqqC* community composition, and microbial interactions as well as weakened the dispersal abilities of *pqqC*-harboring bacteria. To our knowledge, we are the first to systematically investigate the diversity, composition, and assembly mechanism patterns of *pqqC* communities as well as the associated co-occurrence networks along a natural aridity gradient, and these findings could help researchers to predict soil *pqqC* community and its ecological functions respond to ongoing global climate change in semi-arid grassland ecosystems.

## Data availability statement

The data presented in the study are deposited in the NCBI repository (https://www.ncbi.nlm.nih.gov/), accession number PRJNA869305.

## Author contributions

MZ: investigation, sample collection, visualization, and writing. RS, RZ, and XA: investigation and sample collection. GC and HJ: manuscript revision. All authors contributed to the article and approved the submitted version.

## Funding

This work was supported by the Natural Science Foundation of Shaoxing University (20185010), P. R. China.

## Conflict of interest

RZ is employed by Inner Mongolia Autonomous Region Water Conservancy and Hydropower Survey and Design Institute Co., Ltd.

The remaining authors declare that the research was conducted in the absence of any commercial or financial relationships that could be construed as a potential conflict of interest.

## Publisher’s note

All claims expressed in this article are solely those of the authors and do not necessarily represent those of their affiliated organizations, or those of the publisher, the editors and the reviewers. Any product that may be evaluated in this article, or claim that may be made by its manufacturer, is not guaranteed or endorsed by the publisher.

## Footnotes

^1^ https://cgiarcsi.community/
