# Supplementary material for: Soil pqqC-harboring bacterial community response to increasing aridity in semi-arid grassland ecosystems: Diversity, co-occurrence network, and assembly process
Source: Front Microbiol. 2022 Oct 21;13:1019023. doi: 10.3389/fmicb.2022.1019023 (PMC9633997; doi:10.3389/fmicb.2022.1019023)
Supplement: Supplementary file 1 [file Data_Sheet_1.docx]

Supplementary Material

# Supplementary Tables

**Table S1** Description of geographic locations, grassland types, and treatment of all samples in this study.

| Sample ID | Sites | Longitude  (°) | Latitude  (°) | MAP (mm) | MAT  (°C) | Vegetation types | Treatments | Detailed information |
| --- | --- | --- | --- | --- | --- | --- | --- | --- |
| MN_1 | Hulunbuir | 120.049108 | 49.329233 | 303.21 | -1.82 | Meadow steppe | Natural grassland | This sampling site was fenced to exclude grazing or mowing since 2008. |
| MN_2 | Hulunbuir | 120.054785 | 49.331513 | 303.33 | -1.87 | Meadow steppe | Natural grassland |  |
| MN_3 | Hulunbuir | 120.055289 | 49.331807 | 303.33 | -1.87 | Meadow steppe | Natural grassland |  |
| MD_3 | Hulunbuir | 120.054710 | 49.331121 | 303.33 | -1.87 | Meadow steppe | Disturbed grassland | Mowing was conducted at the the end of August every year since 2008. All above-ground biomass was removed above 5cm. |
| MD_4 | Hulunbuir | 120.055407 | 49.331058 | 303.33 | -1.87 | Meadow steppe | Disturbed grassland |  |
| MD_5 | Hulunbuir | 120.056223 | 49.330961 | 303.5 | -1.87 | Meadow steppe | Disturbed grassland |  |
| TN_1 | Xinlinhot | 116.674678 | 43.556142 | 266.46 | 1.58 | Typical steppe | Natural grassland | Fencing enclosure was conducted to exclude grazing or mowing since 2005.  . |
| TN_2 | Xinlinhot | 116.683168 | 43.559151 | 267.5 | 1.55 | Typical steppe | Natural grassland |  |
| TN_3 | Xinlinhot | 116.681945 | 43.559389 | 267.33 | 1.56 | Typical steppe | Natural grassland |  |

**Table S1** Continued

| Sample ID | Sites | Longitude (°) | Latitude (°) | MAP  (mm) | MAT  (°C) | Vegetation types | Treatments | Detailed information |
| --- | --- | --- | --- | --- | --- | --- | --- | --- |
| TD_1 | Xinlinhot | 116.673211 | 43.558478 | 266.81 | 1.57 | Typical steppe | Disturbed grassland | This sampling site were continuously grazed by sheep since 2005. The grazing intensity was 9.0 sheep ha^-1^, and belonged to heavily grazing (Liu et al., 2012). In each year, grazing period commenced at the beginning of June and finished the late of September (lasted for four-month). |
| TD_2 | Xinlinhot | 116.679761 | 43.561669 | 267.08 | 1.57 | Typical steppe | Disturbed grassland |  |
| TD_3 | Xinlinhot | 116.680829 | 43.561816 | 267.08 | 1.57 | Typical steppe | Disturbed grassland |  |
| DN_1 | Siziwang Banner | 111.894196 | 41.790041 | 180.5 | 3.55 | Desert steppe | Natural grassland | This sampling site was fenced to exclude grazing or mowing since 2002. |
| DN_2 | Siziwang Banner | 111.898681 | 41.792417 | 181.25 | 3.53 | Desert steppe | Natural grassland |  |
| DN_3 | Siziwang Banner | 111.903015 | 41.791793 | 181.53 | 3.52 | Desert steppe | Natural grassland |  |
| DD_1 | Siziwang Banner | 111.896374 | 41.792417 | 180.28 | 3.54 | Desert steppe | Disturbed grassland | This sampling site were continuously grazed with a heavy grazing-intensity ( 2.71 sheep ha-1) since 2002 (Liu et al., 2012). Grazing activity started in early June, and finished in the late of November (lasted for six-month). |

**Table S1** Continued

| Sample ID | Sites | Longitude  (°) | Latitude  (°) | MAP  (mm) | MAT  (°C) | Vegetation types | Treatments | Detailed information |
| --- | --- | --- | --- | --- | --- | --- | --- | --- |
| DD_2 | Siziwang Banner | 111.898091 | 41.793009 | 180.33 | 3.53 | Desert steppe | Disturbed grassland | This sampling site were continuously grazed with a heavy grazing-intensity ( 2.71 sheep ha-1) since 2002 (Liu et al., 2012). Grazing activity started in early June, and finished in the late of November (lasted for six-month). |
| DD_3 | Siziwang Banner | 111.906073 | 41.790449 | 182.33 | 3.51 | Desert steppe | Disturbed grassland |  |

**Table S2** Characteristics of climate, aridity, vegetation, and soil physicochemical properties of all samples in this study.

|  | Natural grassland | | |  | Disturbed grassland | | |
| --- | --- | --- | --- | --- | --- | --- | --- |
|  | Low aridity | Medium aridity | High aridity |  | Low aridity | Medium aridity | High aridity |
| Aridity | 0.62±0.00c | 0.74±0.00b | 0.85±0.00a |  | 0.62±0.00c | 0.74±0.00b | 0.85±0.00a |
| Plant richness | 22.00±2.03a | 7.44±1.02b | 6.78±0.69b |  | 7.11±1.39a | 5.56±0.38ab | 5.11±0.51b |
| Plant biomass (g) | 194.91±14.16a | 153.5±15.65b | 72.74±12.61c |  | 207.12±9.35a | 55.21±7.10b | 47.09±2.64b |
| pH | 6.72±0.22b | 7.68±0.24a | 7.97±0.04a |  | 6.54±0.03b | 7.83±0.42a | 8.03±0.04a |
| Soil moisture (%) | 19.44±0.19a | 6.33±0.24c | 7.87±0.37b |  | 16.97±0.68a | 4.59±0.81c | 7.97±0.24b |
| SOC (g kg^-1^) | 32.98±1.56a | 21.72±1.55b | 14.75±0.41c |  | 29.45±0.42a | 18.78±1.55b | 14.69±0.56c |
| TN (g kg^-1^) | 3.01±0.13a | 2.3±0.06b | 1.58±0.10c |  | 2.87±0.04a | 2.09±0.01b | 1.64±0.02c |
| TP (g kg^-1^) | 0.52±0.02a | 0.40±0.02b | 0.38±0.00b |  | 0.47±0.01a | 0.34±0.01c | 0.39±0.00b |
| NH_4_^+^ (mg kg^-1^) | 10.59±1.19a | 3.57±0.38b | 2.25±0.41b |  | 7.91±0.35a | 1.88±0.74b | 1.62±0.30b |
| NO_3_^-^ (mg kg^-1^) | 9.90±1.47a | 6.48±0.88b | 6.91±1.02b |  | 6.78±1.17a | 2.93±0.51b | 5.57±0.92a |
| AP (mg kg^-1^) | 6.11±1.03a | 4.65±0.52a | 5.17±1.15a |  | 5.66±1.09a | 5.01±0.89a | 2.54±0.22b |

Notes: Values are means ± standard error (n = 3). Different lowercase letters indicate significant differences (*P* < 0.05) among different treatments in the same site. SOC, soil organic carbon; TN, total nitrogen; TP, Total phosphorus; AP, available phosphorus.

**Table S3** Topological properties of soil *pqqC* community networks in natural grassland and disturbed grassland.

| Grassland types | Nodes | Edges | Average degree | Clustering coefficent | Modularity | Positive (%) | Negative (%) |
| --- | --- | --- | --- | --- | --- | --- | --- |
| Natural grassland | 206 | 1576 | 15.301 | 0.631 | 0.52 | 77.54 | 22.46 |
| Disturbed grassland | 230 | 4144 | 18.017 | 0.645 | 0.599 | 85.18 | 14.82 |

# Supplementary Figures


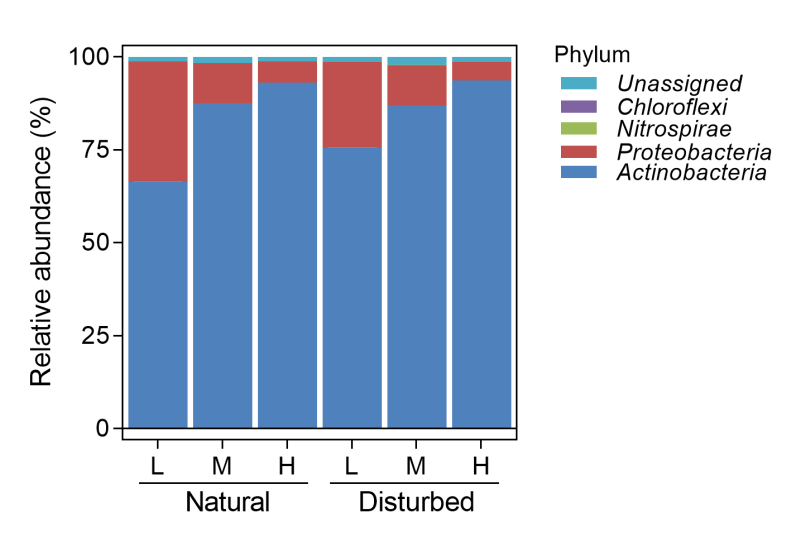


**Figure S1** Variations of relative abundances of *pqqC* taxa at the phyla level under different aridity gradients in natural grassland and disturbed grassland. L, low aridity level; M, medium aridity level; H, high aridity level.

**
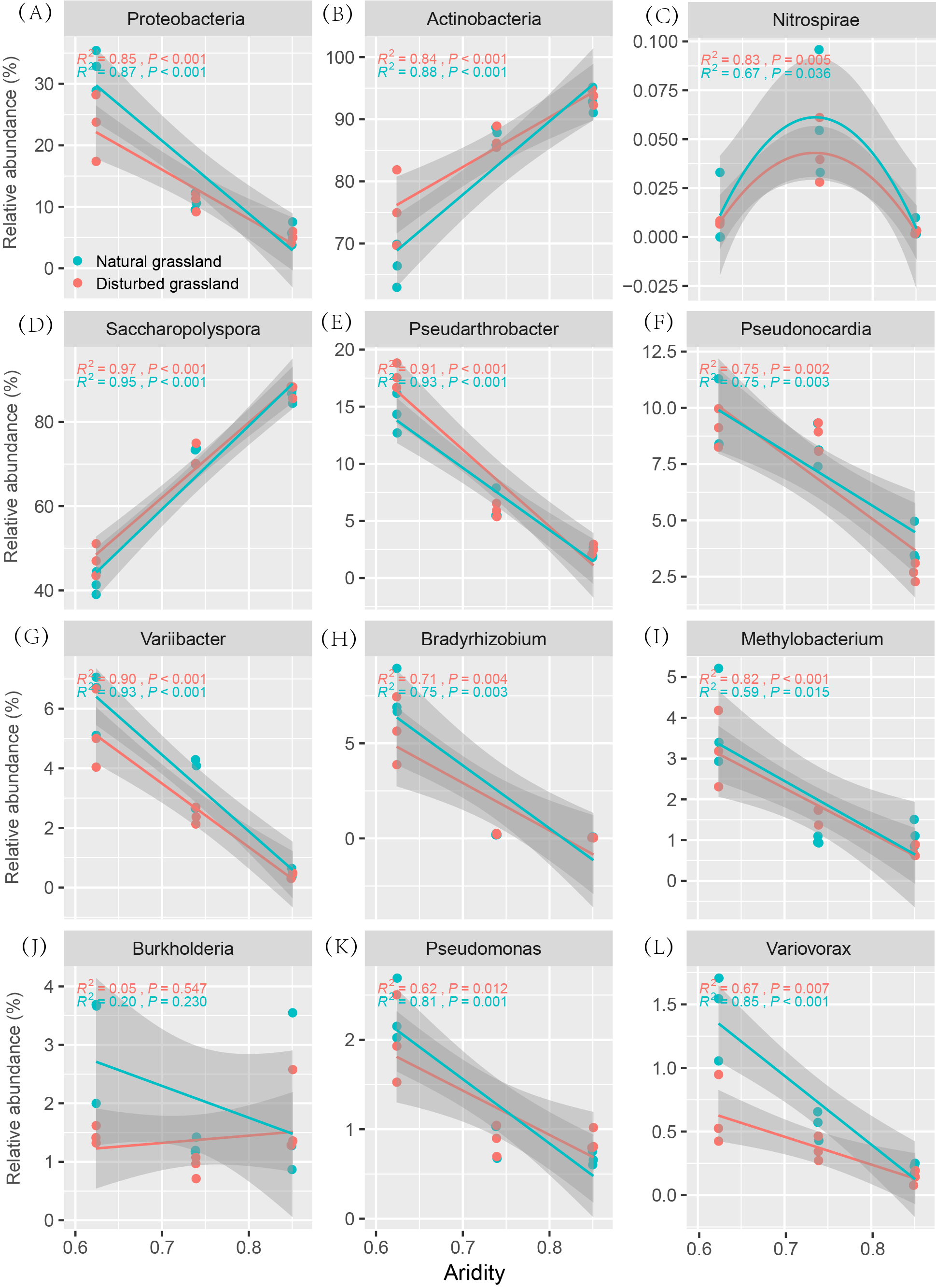
**

**Figure S2** Relationships between aridity and the relative abundances of dominant phylum (A-C) and genera (D-L) of soil *pqqC* community.


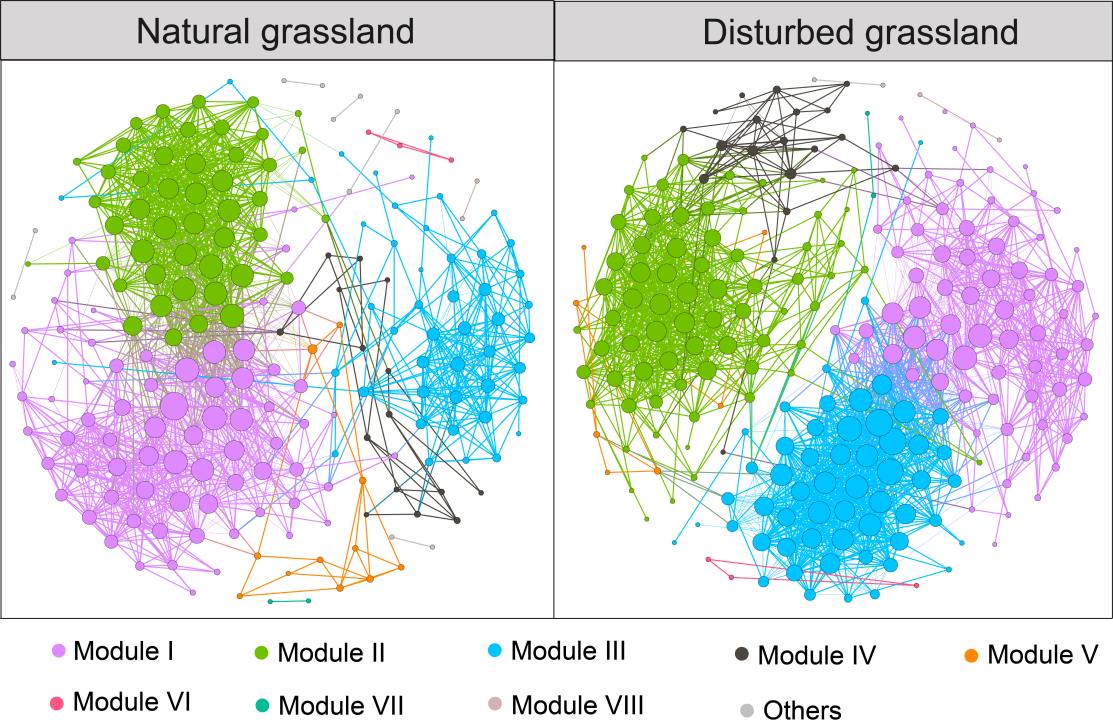


**Figure S3** Co-occurrence networks of *pqqC* community and environmental varibles in natural and disturbed grasslands. Nodes represent individual OTUs; edges represent significant strong and significant Spearman correlations (r > 0.6, *P* < 0.01). All modules are shown in different colors. The size of each node is proportional to the degree.


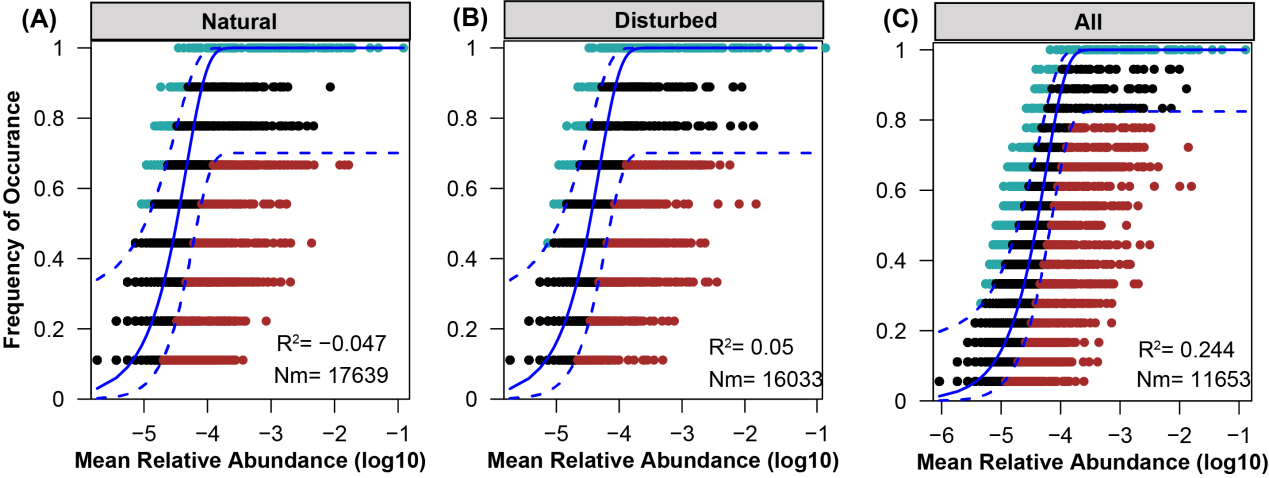


**Figure S4** Fit of the neutral community model (NCM) of *pqqC* community in natural grassland (A), disturbed grassland (B), and All samples (C). The solid blue lines indicate the best fit to the neutral model and the dashed blue lines represent 95% confidence intervals around the model prediction. OTUs that occur more frequently than predicted by the model are shown in aquamarine, while those that occur less frequently than predicted are shown in red. OTUs that occur within prediction are shown in black. Rsqr indicates the goodness of fit to the neutral model. Nm indicates the metacommunity size times immigration.


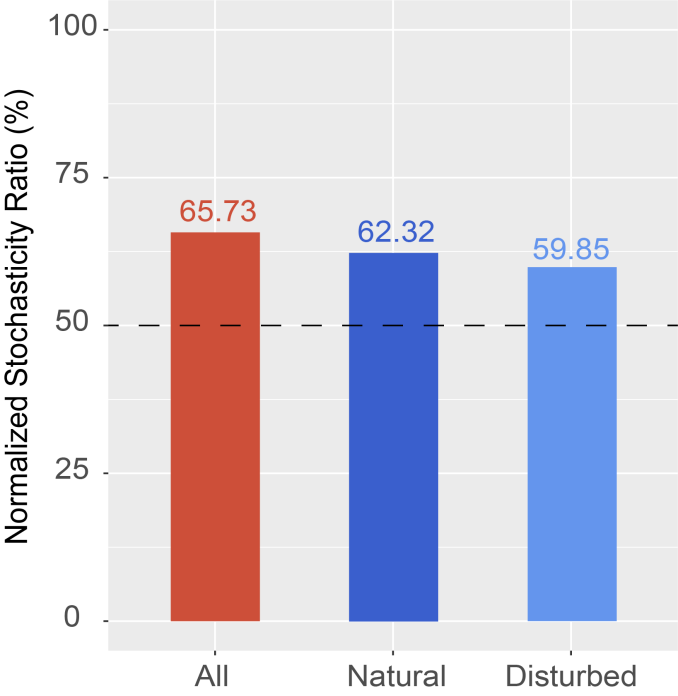


**Figure S5** The normalized stochasticity ratio (NST) of soil *pqqC* community in all samples, natural grassland, and disturbed grassland.


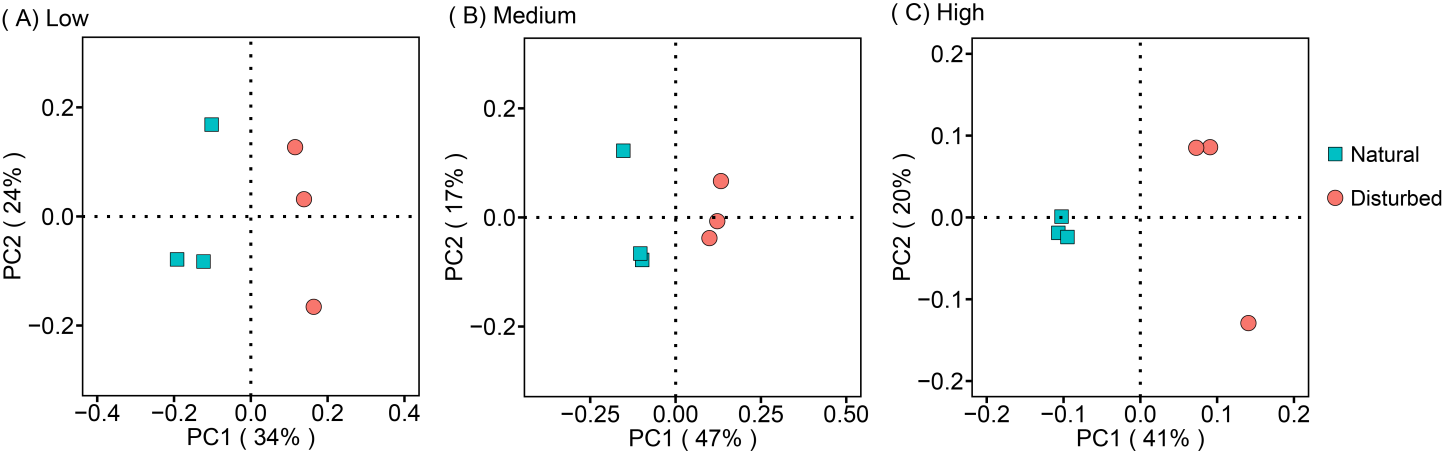


**Figure S6** Principal coordinates analysis (PCoA) of soil *pqqC* community based on the number of OTUs in natural and disturbed grasslands in different aridity levels.


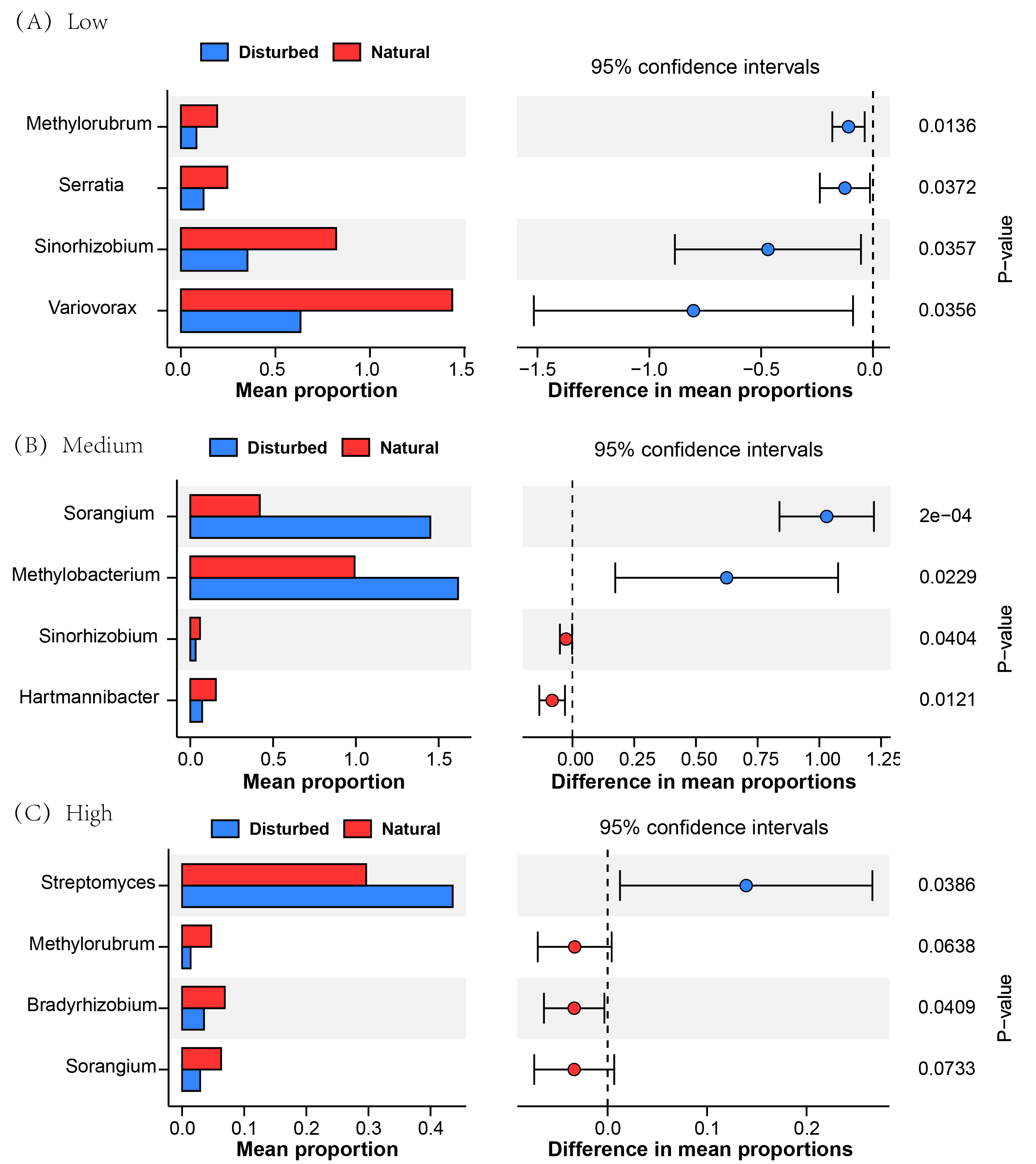


**Figure S7** Changes in the relative abundances of some genus (significant differences) between natural and disturbed grasslands in different aridity gradients.

References

Liu, N., Zhang, Y., Chang, S., Kan, H., & Lin, L. (2012). Impact of grazing on soil carbon and microbial biomass in typical steppe and desert steppe of Inner Mongolia. PLoS one, 7(5), e36434. https://doi.org/10.1371/journal.pone.0036434
